# Diseases of the Windpipe

**Published:** 1838-01-01

**Authors:** 


### II. DISEASES OF THE WINDPIPE.

1. OBSERVATIONS ON THE SURGICAL PATHOLOGY OF THE LARYNX AND TRACHEA, INCLUDING REMARKS ON CROUP, CYNANCHE LARYNGEA, WOUNDS, &c. &c. By *W. H. Porter*, Professor of Surgery in the Royal College of Surgeons in Ireland. 8vo. pp. 275. Second Edition. London, 1837.

2. A TREATISE ON THE DISEASES AND INJURIES OF THE LARYNX AND TRACHEA. By *Frederick Ryland*, Surgeon of the Town Infirmary, Birmingham. 8vo., pp. 328. London, 1837.

3. TRAITÉ PRATIQUE DE LA PHTHISIE LARYNGÉE, &c. P[illegible] MM. *Trousseau* et *Belloc*. 8vo., pp. 488. Paris, 1837. Bail-liere.

THE description of the various morbid changes, to which the larynx and trachea are subject, is, in Mr. Porter's and Mr. Ryland's works, very properly preceded by a brief account of the General Pathology of Mucous Tissues.

Inflammation is, as a matter of course, the first and most important of these changes; and it is always very interesting to trace the varying characters and effects of this most frequent of all diseased actions, according to the part affected, the functions which it exercises, and the sympathies which it has with other systems of the body.

At present, we have no intention of entering upon any minute details of this subject, as such a discussion would necessarily occupy the space which we design to devote rather to therapeutic than to pathological medicine. There is, however, one of the effects, or, as they are usually called, the consequences, of inflammation of the larynx and trachea, which, from its peculiarity, deserves to be noticed—we allude to the effusion of coagulable lymph on their mucous surface in the disease well known under the name of Croup.

Every physician is aware that this most dangerous malady is almost peculiar to infancy and youth. A genuine case of it in the adult is of excessively rare occurrence—so rare indeed, that many have questioned the authenticity of the few examples on record. Certain it is, that the pathologist very seldom meets with real coagulable lymph on any one of the mucous membranes of the body, after an *acute* inflammation of the part, except in subjects under the age of puberty; or, if this position be disputed, at all events this effect of inflammatory action is infinitely more frequent in early than in the more advanced years of life. This remark holds true in reference to the lining not only of the air-passages, but also of the other mucous canals of the body. Thus we occasionally find that inflammation of the alimentary canal causes an effusion of coagulable lymph from its surface; and many cases are recorded of soft shreddy tubular formations having been voided with the alvine dejections. This phenomenon is more frequently observed in the pharynx and œsophagus than in the stomach or any portion of the intestines; and it occurs much oftener in children than in adults.

Such cases, however, do not contradict the truth of the assertion which we have made — that *acute* inflammation of mucous structure very rarely causes an exudation of genuine congulable lymph, except in the trachea only, or, in other words, in the disease of Croup; for it is usually, when the disease of the bowels has assumed a *chronic* form, that the effusion of lymph has been observed to occur. The same remark is applicable to the uterus. The inner surface of this organ is occasionally found to be lined with a layer of membranaceous deposit; but this phenomenon is rarely or never witnessed after acute hysteritis.

There is, however, one form of acute inflammation of mucous tissue—besides Tracheitis or Croup—in which there seems to be an obvious tendency to the effusion of coagulable lymph, and which has of late years, since M. Bretonneau of Tours has written on the subject, attracted the attention of many medical men. We allude to the disease which this physician has designated by the appellation of "Diphthérite" (from διφθερα, pellis, exuvium), or "angine couenneuse." It is, in truth, a variety of Cynanche pharyngea, in which the inflammation manifests a strong disposition to the deposition of a false membrane on every part of the affected mucous tissue. The older writers have mentioned it under the name of *angina membranacea;* and more recently it has been described by some authors as the *pellicular inflammation of the fauces.* As there seems to be not a little incorrectness, in the views of many medical men, on the subject of this malady, we shall devote a page or two to consider some of its more important characters.

We may premise our remarks by suggesting the impropriety of applying a new and special name—that of Diphthérite—to one form only of inflammation of any part or organ. The disease is, in every respect, a mere variety of Cynanche or Angina; and hence it would be far better to affix some epithet, descriptive of its peculiar or pathognomonic features, to these well-known and long-established names, than to increase our nosological nomenclature by unnecessary additions.

Besides this objection, there is a much more important reason for avoiding such new terms. The affixing of a new name to a disease is apt to withdraw the attention of the medical practitioner from the genuine nature of its most important characters, and to direct it, far too exclusively, to the study of one set only of its symptoms. This is the very error into which M. Bretonneau, and those who have adopted his doctrines, have fallen, in reference to the malady at present under our consideration.

It is an error much more common among the Continental than among the British physicians; but even some of our own writers, as we shall afterwards find, have been misled by the circumstance alluded to.

The extreme and, in many respects, exaggerated importance, which of late years has been attached to the study of pathological anatomy, has tended not a little to encourage the prejudice in question. An apt illustration of the truth of this remark may be drawn from the history of other diseases, besides that of Diphthérite. Take, for example, Typhus fever or Hydrocephalus acutus. How vague, fluctuating, and uncertain have been the opinions of many medical men in reference to these two very common and most important maladies! Misled by attending far too indiscriminately to the post-mortem appearances, found on dissection, the French school have vainly attempted to prove that the essential and primary character of Typhus fever is an inflammation of the mucous glands of the bowels, and that all the constitutional symptoms of the disease are merely sympathetic of this morbid condition. Hence the names of *gastro-enteritis, dothinenteritis, fièvre entero-mesenterique*, &c.; and hence the most pernicious practice of invariably applying leeches to the abdomen, and of avoiding the use of purgative medicines, as recommended by so many Continental authors. It is unnecessary to express our sentiments at greater length on these doctrines of the French school, as we exposed their fallacies in our last number, while reviewing the works of M. M. Louis and Bouillaud.

Equally great have been the errors, which have arisen from applying the terms *arachnitis*, or *encephalitis*, to that train of morbid phenomena, which constitute the well-known disease of Hydrocephalus acutus. In making this remark, do not let it be supposed that we believe that this last-mentioned name is quite unobjectionable. Every physician of any experience knows well that numerous are the cases, where all the symptoms of acute Water in the Head have been present during life, and yet on dissection not a tea-spoonful of fluid has been found in any part of the encephalon. The watery effusion is justly considered to be only *one* of the effects of a previous diseased action in the substance of the brain itself. But, while we admit the truth of this, we are by no means prepared to concede to the French pathologists, that this preliminary diseased action is, upon all occasions, a simple inflammatory affection of the brain or of any of its investing membranes; else, why should we not find a hydrocephalic effusion in all fatal cases of phrenitis, or of inflammation of the cerebral membranes, after concussion and other injuries of the head?

These brief remarks will satisfy the judicious physician of the impolicy of altering old-established names of diseases, and more especially of changing them for such as are derived from the *presumed* nature of the morbid action on which they depend, or from one of their more prominent features. It is for this reason that we object to the substitution of such appellations as *gastro-enteritis* for Typhus fever, or *diphthérite* for one only of the varieties of Cynanche.

To prove the impolicy of such a nomenclature in reference to the latter disease, we shall now shew our readers what confused, incorrect, and practically dangerous doctrines are announced in some of the most recent works on diseases of the air-passages.

Let us first hear what meaning M. Bretonneau and his followers attach to the term Diphthérite, and what morbid states are designated by it. Mr. Ryland says:

"This name has been given, by Dr. Bretonneau of Tours, to that species of croup which affects adults. It is a disease of much rarer occurrence than the one that has just been described, and possesses characters sufficiently distinctive to entitle it to a separate notice. At the same time, it is proper to state that Bretonneau considers that the ordinary croup of children, the cynanche maligna, and the plastic angina, or *angine couenneuse* of the French, are all precisely the same disease, but producing different effects according to the seat and degree of the morbid action."

His description of its symptoms is as follows:—

"The diphthérite commences with pain in the throat, difficulty of swallowing, and general febrile symptoms; on inspecting the fauces, the tonsils are observed to be swelled and reddened, and to have upon their surface patches of a thick, opake, whitish concretion, which at this period of the disease are easily detached from the mucous membrane. If allowed to go on unchecked, the inflammation and the membranous exudation spread by continuity to the adjacent parts, the soft palate and pharynx; the glands at the angles of the jaw begin to swell, and deglutition becomes more difficult. If the concretion be detached from the membrane beneath, the redness greatly augments in the denuded parts, and another and thicker concretion is soon poured out, which adheres to the mucous surface with more tenacity than before. It frequently happens, that some days after the commencement of the attack, the disease becomes milder in character, less disposed to spread, and even ceases altogether without reaching the air-passages, in which case there is very little reason to be under any apprehension for the result. In most instances, however, at the end of four or five days, laryngeal symptoms begin to display themselves, such as a hoarse cough, alteration of the sound of the voice, and dyspnœa. From this time the patient has every appearance of suffering from severe croup, with the addition of an almost complete inability to swallow; the breathing becomes quick, laborious, and sonorous, the voice is soon extinct, the countenance livid, and the pulse small and intermitting; paroxysms of suffocation take place, till in one more severe than the rest death closes the scene."*

The extraordinary doctrine announced in these extracts—a doctrine which teaches that Croup and Cynanche maligna are diseases of the same nature, varying only according to the seat of the affection and the constitution of the patient—will not fail to astonish the reader, who is not in the habit of perusing the medical literature of the Continent; and yet, strange to say, such men as Laennec and Guersent, one of the most experienced practitioners in Paris at the present time, and a physician to the Hôpital des Enfans, appear to have adopted it to its full extent. The following passage, from Laennec's great work, will show what an erroneous view he took of the question.

"Sometimes *croup* is confined to the bronchi and their branches, there being no trace of it in the larynx and trachea. More commonly however, as has been shewn by Bretonneau, the inflammation commences on the tonsils or the pharynx, and from thence spreads at the same time downwards to the larynx and upwards to the cavity of the nostrils, which latter it sometimes entirely covers. Occasionally, the false membrane extends to the stomach." Guersent thus expresses himself as to the analogy between croup and putrid sore throat: "M. Bretonneau has demonstrated that the epidemic malignant angina is a genuine pellicular inflammation, similar to that of croup. He has also proved that these two diseases, identical as to pathological changes, differ from each other only in respect of the seat, which they occupy."

We are glad that such able writers as Dr. Cheyne of Dublin, and Dr. Tweedie of London, have, in their contributions to the Cyclopædia of Medicine, exposed the fallacies and errors, into which the modern French

* A shorter definition of the disease may be desirable. We may therefore state that by the term Diphthérite the French pathologists understand every form or species of recent disease of the fauces, pharynx, and upper portion of the air-tubes, in which the affected mucous surface is covered with a membraniform or pellicular exsudation; whether this be the result of active inflammation, as in croup and some cases of laryngitis, or of that putrescent action which exists in cynanche maligna and in certain epidemics of measles and scarlatina.

pathologists have fallen upon this important practical question. They have most clearly shewn that, under the term Diphthérite, the most dissimilar diseases are grouped together—diseases which have no features of analogy, the one to the other, except in the mere circumstance of their affecting generally the adjacent, but sometimes the same, parts of the body.

The seat of genuine Croup is almost always limited to the trachea and lower portion of the larynx. The disease commences there, and very rarely do we ever see the slightest mark of disease in the pharynx or any part of the fauces. It is essentially sthenic, and highly inflammatory in its nature, requiring for its treatment the most active antiphlogistic remedies. It is peculiar to infancy and youth, and is perhaps never observed after the age of puberty. It is never contagious, and is even seldom epidemic; most cases being clearly traceable to exposure to cold and damp. Such are some of the essential characters of genuine croup.

To attempt to give any exact idea of the real nature of Diphthérite is almost impossible; seeing that various affections, of the most dissimilar attributes, are grouped together under this term. We have already stated that its primary and essential feature is the exsudation of an albuminous, coagulable effusion on the mucous surface of the fauces and air-passages; and that this effusion almost always commences on the velum palati, tonsils, and pharynx, extending from thence along the nares, and sometimes down into the larynx and trachea, and even along the œsophagus into the stomach. But this effusion has no features which are uniform and permanent. Sometimes it is a firm lymph-like deposition, and is the result of an active inflammation of the fauces, as in Cynanche pharyngea and tonsillaris. At other times, it is soft, shreddy, in patches, like sloughy mucous membrane, and its presence is accompanied with all the symptoms of putrid typhus fever, as in Cynanche maligna, and in some of the worst cases of scarlatina and of measles.

These two opposite forms of disease have no common features of resemblance, the one to the other, except in the mere circumstance of the same parts being affected. The one is sthenic—the other is asthenic, with a decided tendency to putridity; the one may, or may not, be epidemic and contagious, the other is almost invariably so; the one requires an antiphlogistic treatment, the other is, as a matter of course, invariably aggravated by such remedies, and must be combated by cordials, antiseptics, and so forth. In short the two forms of disease, now alluded to, are as distinct from each other, as Synochus is from Typhus gravior. Dr. Tweedie very properly prefers the generic appellation of Angina or Cynanchea membranacea to that of Diphthérite; distinguishing the one form, as the acute, and the other as the malignant or putrid.

There are various shades or degrees of both forms, according to the violence of the sthenic or asthenic symptoms, and to the extent of the membraniform effusion. As long as this effusion is limited to the fauces and pharynx, there cannot be any resemblance, in either form, to the peculiarly characteristic signs of genuine croup—*inspiratio strepens, vox rauca, tussis clangosa*, the shrill ringing cough followed by a deep crowing sonorous inspiration, and more or less complete hoarseness of the voice; but when it spreads to the larynx and trachea, these symptoms, as might be expected, to a certain degree supervene.

This complication is much more common in the course of the acute, than in that of the malignant, form of the Angina membranacea. We observe it not unfrequently in some epidemics of scarlatina, measles, and small-pox, in which the throat symptoms are unusually violent. The fauces, during the early stage of the complaint, are found to be uniformly red and inflamed, often of a somewhat mottled complexion, in consequence of certain points being of a darker hue than the rest of the vascular surface.

To this state succeeds the appearance of numerous small white or greyish spots, which, at first detached and isolated, gradually enlarge in extent, until they finally coalesce and form a pellicular covering over the entire surface.

MM. Brettonneau and Guersent have traced, on dissection, this effusion along all the passages which communicate with the fauces; upwards as far as the Eustachian tubes and frontal sinuses, and downwards into the trachea and the œsophagus. The subjacent mucous membrane seldom exhibits any morbid appearance, save that of extreme vascularity: in a very few instances it has been found slightly eroded. Even in the malignant form of the disease, the Cynanche maligna of most British writers, the integrity of the mucous tissue itself has been rarely, according to the pathological researches of the French physicians, detected to be at all injured.

What were formerly supposed to be sloughs of the mucous membrane have been proved to be, in most cases at least, pellicular membranes or exsudations. "When these are tinged with blood," says Dr. Tweedie, "and especially when at the same time blood exudes from the lips and gums, their dark or almost black appearance and intolerable fætor have frequently caused them to be mistaken for sloughs; the surface, however, from which they are detached is perfectly entire, the mucous membrane, although of a dark red or livid colour, is perhaps not even softened, nor is there any peculiar odour of gangrene."

We willingly concede to M. Bretonneau the merit of having first clearly demonstrated by careful dissection the real pathology of this disease, the Cynanche maligna, or, if you will, the malignant form of Diphthérite, or Angina membranacea; and, had he been more cautious and circumspect in his conclusions, and less hasty in generalising facts and phenomena, of the most dissimilar character, under one common description, this merit would have been tenfold better deserved.

Let it not be supposed that the question, which we have been canvassing, is one of mere speculative moment, and that it does not affect the doctrines of practice. We have only to examine the therapeutic instructions in the works of M. Bretonneau and of other French physicians on the subject of croup, to be satisfied of the errors, which their recent speculations about the analogy between this disease and cynanche maligna, have led them into. Before, however, doing this, it may be useful briefly to describe the practice which is recommended by the best physicians in our own country, and to the value of which we can testify from the results of a pretty extensive experience.

The *first* thing to be done, when we are called to a case of Croup, is to administer an emetic, in order to ensure a full and powerful action on the system, independently of its merely evacuating effects. For this purpose, by far the best is a mixture of the tartrate of antimony and of ipecacuan powder. The formula, which we generally use, is a couple of grains of the former and a scruple of the latter to an ounce and a half of dill-water, sweetened with half an ounce of simple syrup. A dessert-spoonful or so—according to the age and strength of the child—of this mixture may be given every ten minutes until copious vomiting be induced. Such is the simple and efficacious remedy, which is first to be employed.

When once the desired effects are obtained, the physician ought, without delay, to abstract a certain portion of blood. This is to be done by means either of venæsection or of leeches. Whenever the case is, or threatens to be, violent, the former is decidedly to be preferred, however young and apparently feeble the little patient may be. The object is, not merely to withdraw a certain portion of blood, but to make a speedy and decided impression on the general powers of the system, so as to keep up and to increase the effects already procured from the administration of the emetic. Often indeed there is extreme difficulty in opening a vein in a young child, especially when he is fat; the subcutaneous vessels being completely hid among the dense, firm adipose tissue. Still, however, let the trial be made: a mere incision or two through the skin are of no consideration, when the very life of our patient is at stake. If no vein of the arm can be found, let the jugular vein or temporal artery be tried; or perhaps some of the veins of the foot will be found to afford a small quantity of blood. Some physicians, indeed, have recommended that the jugular vein should *on all occasions* be preferred to any of those on the arm, even when the latter are quite distinct, and may therefore be readily opened. On this point we do not agree with them. The bleeding is seldom or never so free from the jugular vein, in consequence not only of the looseness of the integuments of the neck, but also of the movements of the head, which the child is almost constantly rolling about from one side to the other. It is a mistake, we believe, to suppose that the withdrawal of blood from a vessel in the neighbourhood of the suffering organ is much more, if indeed at all, efficacious in arresting inflammatory action, than from a more distant one.

If a sufficient quantity of blood can be obtained to induce a decided depression of the vital powers, it matters not whence it is drawn; and that method is to be preferred which least incommodes or distresses the patient.

Supposing, however, that we do not succeed in drawing blood enough by venæsection, or, again, if the disease is not so violent as to require it, the physician should then trust to local bleeding by means of leeches; and, in reference to this point of practice, there are two *hints* which we wish to impress strongly on the mind of the young practitioner. The first of these is that he will do well, in a case of such extreme urgency as that of Croup, to act the part of *nurse* as well as of *doctor*. Let him, therefore, not entrust the application of leeches to any of the attendants, but do it himself, or have it done by a professional assistant. There are such numerous mistakes constantly made, even by experienced nurses, in using this apparently so simple a remedy—either by applying the leeches in the wrong place, or teasing and distressing the patient by frequent ineffectual attempts—that we are confident that its good effects are often lost through their awkwardness and ignorance. Independently of the high principles of duty—and few duties are more imperative than those which are very properly required of all, who are entrusted with the lives of their fellow-creatures—as well as of professional curiosity to watch the effects of his practice, the physician will often have the pleasing delight of receiving the unmingled gratitude of affectionate parents to reward him for an hour or two's attendance. Of this, at least, he may be assured, that he will never have cause to regret his trouble, however large may be his practice, and established his fame.

The second suggestion, which deserves our attention, is respecting the proper place where the leeches are to be applied. As a general remark, we may assure our younger professional brethren that leeches should seldom or never be applied, at least in any considerable number, along the front of the neck of an infant. Not only is there frequently the greatest difficulty experienced in checking the hæmorrhage from this part, but the distress and inconvenience to the little sufferer, from the irritation excited as well by the leech-bites themselves as by the often-repeated attempts to apply the leeches in the first instance over the very seat of the disease, far outweigh any supposed advantages of drawing the blood from the immediate neighbourhood of the affected organ. Every good effect may be obtained from putting on a sufficient number of them, over the upper part of the chest, along the top of the sternum and sternal ends of the clavicles. The simplest plan is to apply a wine-glass, containing from half-a-dozen to a dozen leeches over the sternum. If more are required, we have only to make a second application. In this way, the bleeding points are kept all near to each other; and thus the effects of the continual oozing are much more decided and effectual. The oozing is readily maintained, for almost any length of time, by laying a thin piece of rag, dipped in hot water, over the bleeding surface; and is easily checked, when this is deemed necessary, by slight pressure with the finger upon the subjacent bone.

These *hints* will not be deemed unnecessary by those who have seen many cases of croup treated, either in their own practice, or in that of others; and we are the more disposed to inculcate their importance, from the omission of all advice on the subject in almost every recent work, which we have looked into. For example, Mr. Ryland seems not to be at all aware of the inconveniences, not to say positive disadvantages, and even the occasional danger, of applying a number of leeches to an infant's throat.

"In the majority of cases, the application of leeches *along the course of the trachea* will be preferable to general blood-letting, because the disease more frequently occurs in weakly children than in those that are habitually healthy; but when the febrile excitement is great, the face flushed, and the respiration and pulse much quickened, venesection to the extent of producing faintness should be put in practice, for this disease has many strong points of resemblance to inflammations of the serous membranes, and in such complaints the advantages of a full and early bleeding are universally allowed."* 146.

---

* The author, in a subsequent page, has certainly undervalued the good effects of bleeding in poor practice. From the results of eight cases—in four of which the patients were leeched and died, and in the other four they were not and yet recovered—"we may," says he, "I conceive, draw an inference unfavourable to bleeding in croup amongst the paupers of a large town."

Be it remembered, that in every one of these, eight cases, "the complaint had been overlooked and neglected, and relief was sought at a very late period."

Under such circumstances as these, indeed, the omission of bleeding may be quite proper. It is only to the universality of the advice that we make any objection.

And, on the other hand, Mr. Porter, while he differs from Mr. Ryland as to the comparative advantages of general and local bleeding in Croup, has most assuredly undervalued the great importance of the latter method, and has exaggerated its inconveniences, in consequence of neglecting those very hints, which we have alluded to above. The following passage will satisfy our readers of what we mean.

"Bloodletting, in croup, should be always general, because the object is to produce direct and immediate debility, and the arm or the external jugular vein may be indifferently chosen for the purpose. Topical bleeding by leeches, &c., may be resorted to in cases where, in consequence of the child being very fat, it may be difficult to discover a vein, but otherwise I regard this practice as highly objectionable. It does not answer the purpose of immediately reducing the patient; and it is questionable whether it can produce any effect on the vessels of a part which lies so far removed from the surface. The oozing of the blood renders the condition of the patient uncomfortable and filthy. The hæmorrhage is often so difficult to be controlled as to render bandages and compresses necessary; and these, applied to the neck, never fail to aggravate the sufferings of a patient already perhaps in a state of suffocation. But what is more important, it is often absolutely impossible to stop the bleeding from leech-bites on children, and many have perished from this cause alone. Has not every practitioner seen children pale and exsanguineous, with heaps of rags and flour and lint piled over the punctures in order to stop the blood, which still continued to trickle, notwithstanding the weakness of the little patient, until the powers of life become irrecoverably impaired? Therefore, in all cases where it may be practicable, I would prefer general bloodletting; and this should be followed by the exhibition of some preparation of antimony calculated to produce a state of nausea, and no more." 31.

We now proceed to the other parts of the treatment. No sooner has the emetic acted freely, and a sufficient quantity of blood been drawn, (or, if leeches have been applied, while the oozing still continues,) than we should commence the use of mercurials, given in such doses as to affect the system. For this purpose, we may order a grain or more of calomel, combined with the same quantity of James's powder, and of the nitrate of potass, every two hours, alternating each dose with a table-spoonful of a solution of tartar-emetic, or of a mixture of ipecacuan powder, so as to keep up a constant nausea.

In this manner, we shall very often succeed in making a speedy impression on the malady; as no hour passes over, without either the calomel, or the nauseant being administered. Should our patient be very feeble, or should there be a disposition to diarrhœa, the hydrargyrum cum cretâ may be substituted for the more active calomel, and the ipecacuan, in repeated doses of one or two grains, may be substituted for the antimonial preparation.

With respect to the effects of the mercurial treatment of croup, Mr. Porter does not award to it that importance which, in our opinion, it deserves. He has said:

"The exhibition of mercury has also had its advocates in the treatment of croup, and within my own experience it has appeared occasionally to have produced material benefit; but its use is nearly confined to long-protracted and chronic cases, where there is a tendency in the mucous membrane to become thickened and changed in its structure. Sometimes, in large doses, it has seemed to have been useful even in the commencement of acute attacks, although either here or in more chronic affections it is not easy to explain its mode of operation. It is very rarely that calomel, however administered, produces any sensible effect on children under nine or ten years of age, except when it exhibits its purgative effects; but something else must be looked for with reference to its operation in the cure of croup, for certainly it accomplishes more than could be expected from any other purgative whatever." 35.

His remarks however on the use of blisters in this disease so completely coincide with our own, and are so thoroughly practical that we have great pleasure in submitting them entire to the reader's attention.

"It is always hazardous to apply a blister in the immediate neighbourhood of inflammation, and particularly so if the constitution has not previously been brought down by bleeding and other evacuants, lest it should increase the evil it is intended to remedy. But a consideration of more importance is, that patients who have laboured under difficult respiration for any time always have their vital energies impaired. In some instances, a blister has seemed to be perfectly inert—has never risen, or even produced a rubefacient effect: in others the consequences have been truly formidable, deep and extensive sloughs having formed wherever the blister had been applied. In the more advanced stages of croup, when the lungs are congested and there is a tendency to effusion within them, there seems to be less objection to the use of this remedy as applicable to the chest itself. But, after all, where is the value of removing this congestion, if it was even in our power to do so, without at the same time relieving the obstruction to the respiration which occasioned it? I have generally found the application of a blister to the chest in this disease to be nearly useless; when applied to the throat, it has but too frequently proved worse." 14.

It is scarcely necessary to observe that our objections to the use of blisters in croup apply to the *earlier* stages only of the disease. When the active phlogistic symptoms are once arrested, and when there is a tendency, so to speak, to recovery, decided benefit may be derived from the application of a blister to the upper part of the chest, or sometimes to the nape of the neck.

There is another class of medicines, to which we have not yet alluded, but the use of which should never be omitted, unless certain contra-indicating symptoms chance to exist: we refer to that of purgatives. Every physician knows the importance of free and repeated evacuations of the bowels in all febrile diseases. Their good effects are attributable not merely to the discharge of all offensive matters from the intestines, but also to the powerful derivation which they effect upon distant suffering organs, and to the general depression which they produce. In such diseases as Croup—in which it is necessary to be administering to a child medicines so frequently as every hour or two—enemata are by far the best mode of soliciting the action of the bowels. A purgative enema may be given every four or six hours, without any inconvenience to the infant, or in any way interfering with the other parts of our treatment.

Even in cases, where there is a disposition to diarrhœa, we have derived much good from the use of injections—not purgative indeed, but simply emollient, or of gruel or barley-water with or without the addition of oil and moist sugar. Besides the soothing effects not only upon the bowels, but on the whole system, they in some degree prevent the irritating effects which the large doses of mercurial and antimonial preparations are so apt to occasion.

In Croup, as indeed in all other diseases of violent excitement, there is a *period* at which the use of antiphlogistic and depressing remedies must be discontinued, and recourse should be had to quieting and even to slightly stimulating measures.

When the pulse becomes soft and easily compressible, when there is any, even the slightest, tendency to syncope, when the breathing is rapid and yet not deep, and when the surface loses its feverish heat, it is time to make a change in our practice. The administration of weak beef or chicken tea is the safest and best stimulant, which we can give at first. Along with this, a few drops of sal volatile, with minute doses of opium or of hyosciamus, in a mucilaginous mixture, may be administered frequently; and all evacuations from the bowels should be moderated or repressed.

When the symptoms of exhaustion are more decided, stronger and more frequently repeated stimulants must be employed. Of these burnt brandy, æther, and ammonia, with or without opium, are the best. Under such circumstances, it is always a most important object to procure sleep—hence the gratifying results, in many cases, from the judicious exhibition of opiates at the proper time.

Before dismissing the treatment of croup, it is right that we should allude to the proposal of performing tracheotomy, when other means seem to fail. In this country at least, the operation has been of late years certainly, and we think quite deservedly, falling into discouragement. The following extracts from Mr. Porter's work are, in our opinion, quite expressive of the sentiments of most British practitioners.

" Quite independently of the difficulty of performing the operation of bronchotomy on a child, which forms, however, a most material objection, it is very doubtful whether it will ever become a favourite remedy in croup. Dr. Cheyne, in his work on the Pathology of the Larynx and Bronchiæ, states that there are always ³ths of the canal free for the transmission of air, a space which would be sufficient to maintain the process of respiration even in an adult subject. It would appear, therefore, that the patient dies, not because there is an absolute insufficiency of air to provide for the arterialization of the blood, but because some change has taken place in the organ by which this most important function is performed. In all cases where the disease consists of inflammation of the mucous membrane I feel satisfied that the artificial admission of any quantity of air (which is all that can be accomplished by the most successful operation) will confer no benefit on the patient beyond relieving him from the exertions he is obliged to make to dilate the rima glottidis,—exertions which he will make if the larynx be only partially closed, and a sufficient aperture for the passage of the air still remains." 47.

Having thus described the treatment of Croup, which we have found most efficacious in our own practice, and which is approved of by most British writers of repute, let us briefly compare it with that, which is recommended by some of the most recent French authors.

We formerly stated that the strange hypothesis of identifying Croup with the other forms of pellicular or membranaceous angina, including Cynanche maligna, has necessarily led its advocates into some pernicious errors in a practical point of view. For example, M. Laennec remarks :—

" All practitioners, who have had occasion to see a good deal of this disease (of croup) will readily admit that these (antiphlogistic) measures, although very rational and conformable to the results of experience in the treatment of inflammatory diseases in general, are nevertheless rarely sufficient, and that very few well characterised instances have yielded to their influence."

And M. Bretonneau uses language still more emphatic on this subject:—

"I am forced to declare, contrary to the received opinion, that bleeding in croup has done harm, and accelerated rather than retarded the spreading of the coriaceous inflammation. I did not abandon this measure, till after repeated proofs of its injurious effects."

Such advice can require no comment, when addressing the British reader. If these authors allude to genuine Croup—the angina or cynanche trachealis of the older writers—we need scarcely say that the treatment, which they recommend, deserves every reprobation; and, on the other hand, if they are alluding to the Cynanche maligna and some other forms of sore throat, which we occasionally meet with in certain unfavourable epidemics of scarlatina, measles, small-pox, &c., their language, in applying the term *Croup* to such affections, is most censurable.

We might, probably, have not insisted so much on the errors of the French school, if we had not perceived the tendency of some writers in this country to be misled by their novelties. Mr. Ryland, while he avoids the extravagant folly of regarding Croup and Cynanche maligna, as analogous diseases, has in his chapter on *diphthérite*—(a term which, as we have already stated, is quite unnecessary in medical nomenclature)—made use of language which seems to us to be certainly objectionable. For example, he says:

"If we compare the symptoms and the pathological effects of the diphthérite with those observed in the ordinary forms of infantile croup, we are struck at once by the many points of analogy that exist between them, though there are some differences in their origin and progress. That the two diseases are similar in their nature can admit of no doubt; the latter periods of both affections are precisely the same, the organic lesions in the air-tubes discovered after death are also exactly of the same character." 171.

That there is one form of Diphthérite or Angina membranacea of a decidedly inflammatory nature, and which therefore may be said to be, similar in its nature to Croup, is very true; but Mr. Ryland seems to have forgotten that there is another form, we mean the genuine Cynanche maligna, of a very different character, and whose nature has no analogy with it.

Indeed the whole of the chapter on Diphthérite in his work requires revision, if not indeed exclusion, at least under this appellation. The author throughout, labours to give a description of a disease, on which he is far from having any clear notions. He trusts much more to what M. Bretonneau, or what M. Louis has written than to his own experience and observation. Even on the subject of the treatment, he is evidently not *at home.* For example, he says, or at least implies, that as Diphthérite—which, be it remembered, includes Cynanche maligna—is, at its commencement, so much a local disease that the constitutional symptoms might almost pass unnoticed, and as it only becomes dangerous by spreading from the fauces to the air-tubes, our chief reliance should be placed in the application of topical remedies, such as the muriatic acid, a solution of nitrate of silver, or of alum, &c., to the inside of the throat. The only constitutional remedy he mentions as being "not quite destitute of efficacy" is mercury.

We need scarcely say, that in numerous cases of Diphthérite or Angina membranacea, there are other remedies far more safe and valuable than mercury, and that this drug is often positively injurious, if employed otherwise than as a purgative. The exhibition of effervescing salines and of stimulants and cordials, along with the use of gargles, or the direct application, in certain cases, of the lunar caustic, or of a strong acid to the fauces, is a much more rational, and will be found to be a much more successful, mode of practice than that of giving large and repeated doses of calomel.

On the other hand, in the acute form of Diphthérite, local bleeding and nauseants are not to be neglected, however useful the mercurial treatment may be at the same time.

But it is now high time to quit the subject of Croup and Diphthérite.

We propose to devote the remaining few pages of this article to the brief consideration of that most distressing chronic disease of the larynx, which has been called Phthisis laryngea.

It was at first our intention to have examined the history of phthisis laryngea at considerable length, and for this purpose we had prepared a summary of the most important facts and observations recorded in the works of MM. Trousseau and Belloc, and of Mr. Porter, on the subject. The extent, however, which the present article has already reached, will prevent us from doing more than merely calling the attention of the reader to one or two of the more prominent topics; and we shall reserve the minute details till our *next number*, when we hope to complete our review of the pathology of the larynx and trachea.

Under the term of Phthisis laryngea are usually included all organic alterations of the upper portion of the windpipe, which are accompanied with cough, dyspnœa, purulent or bloody expectoration, and the symptoms of hectic fever.

It has been a question among some pathologists whether ulceration of the larynx or Phthisis laryngea is ever a primary idiopathic affection, or whether it is not always secondary to disease either of the lungs or of the fauces. That the windpipe is very frequently involved in the general morbid changes which attend advanced *phthisis pulmonalis*, is known to every physician, more especially since the admirable pathological researches of M. Louis. From these it clearly appears that, the larynx suffers in nearly one-third or one-fourth of those who die from genuine consumption. But it is comparatively a rare occurrence to meet with a case, in which the ulcerative action has commenced primarily in this organ, anteriorly to any affection either of the lungs themselves or of the fauces.

MM. Trousseau and Belloc, in treating of the causes of Phthisis laryngea, admit four species of the disease; according as it is the consequence of— 1. Simple or scrofulous chronic laryngitis; 2, Of syphilitic cachexia; 3, Of scirrhous and cancerous action, and 4, Of the tubercular affection of the system. The symptoms are nearly alike in all these varieties of the disease, and may be briefly described as follows.

The earliest one, which usually attracts notice, is an alteration in the tone of the voice. Sometimes it becomes only weaker than before; at other times it is simultaneously very rough and hoarse, the hoarseness being always aggravated by any fatigue or by vicissitudes of temperature. At first these changes are inconsiderable and intermittent; but as the disease advances, they are more conspicuous, and ultimately become quite permanent. Along with the affection of the voice, there is almost always a cough, which varies much in severity and character in different cases, and more especially in the different varieties of laryngeal disease. It is very generally accompanied with the expectoration of sputa, which are at first mucous and transparent, and afterwards are purulent and very fœtid. Pain too is a symptom which is usually present; but it must be confessed that this is one, on which we cannot place much reliance. In certain cases the patient complains little, if at all, throughout the whole course of the disease; in others it is sharp and troublesome at the commencement and during its earlier stages, and ceases altogether afterwards; whereas, in a third set, it continues throughout the whole course of the disease. The pain, it is most worthy of notice, is almost always much more aggravated by the act of swallowing than by any other effort, except when firm pressure is made on the outside of the larynx. It is, however, to be remembered that, in not a few examples, even swallowing does not produce any inconvenience.

In the more advanced stages of the disease, the physician may sometimes receive partial assistance in his diagnosis from a careful inspection of the fauces. The uvula and tonsils are often found ulcerated; and, occasionally, even the surface of the epiglottis is similarly affected, and may be perceived, when the tongue is well protruded from the mouth. It might be supposed that, when the larynx is seriously affected, as when its cartilages are carious or necrosed, the integuments of the throat might be swollen and painful. But this is rarely found to be the case, at least in the Simple laryngeal Phthisis, according to the experience of MM. Trousseau and Belloc. Certain authors have told us that, in some cases, they have met with a very distinct crepitation, when the larynx was moved about under the fingers; and this symptom they have attributed to the rubbing of the necrosed and partially detached portions of the cartilages, the one against the other; but, independently of the extreme rarity of the occurrence, it is to be observed that the same sound is sometimes to be heard even in a state of health.

The most characteristic symptoms, after those furnished by the changes of the voice, are those connected with respiration. In the early stage of the disease, the breathing is little, if at all, disturbed, except perhaps on active exercise. Then the act of inspiration is usually accompanied with a crowing or piping sound, the act of expiration being free from this phenomenon.

As the disease advances, the breathing becomes more panting and oppressive, although the lungs may remain all the while unaffected by the morbid change. The character of the dyspnœa is somewhat different from that, which is usually present in pulmonary consumption. It is more paroxysmal; the patient being every now and and then subject to the most distressing fits of asthmatic distress. These fits are always the most frequent and most severe during the night; and, as might be expected, they are much influenced by the vicissitudes of weather, &c. The agony of these attacks is often most appalling; the poor patient gasping and writhing ineffectually for breath, like a strangled man. In some cases, death is the immediate consequence of such an attack.*

* Our French authors have canvassed at some length the question whether the trachea or the bronchi are endowed with any share of contractility, as some authors have supposed. They answer in the negative; and refer the more violent symptoms of asthma, croup, &c., to a contracted state of the rima glottidis, and not to any spasm of the alleged muscular coat of the air-tubes.

We have already alluded to the difficulty of deglutition, as one of the symptoms of Phthisis laryngea. In some cases, the difficulty is so great that the patient cannot swallow any solid food without the most urgent distress; and, when he attempts to swallow a liquid, a portion of it escapes into the larynx and causes a violent fit of coughing.

It was formerly supposed that, when such a state of things was present, there was always an actual destruction of the epiglottis. But this supposition is now acknowledged to be erroneous; and we have the authority not only of MM. Trosseau and Belloc, but also of all the best pathologists of the present day, that, on the one hand, an extreme degree of *dysphagia* may exist, when the epiglottis is quite sound and entire; and, on the other hand, that the act of deglutition is occasionally but little incommoded in cases, in which, on dissection, this valve has been found to be almost entirely destroyed.

The symptoms, which we have above described, are applicable to all the different forms of Laryngeal Phthisis. But each form or species of the disease may present certain special phenomena, attention to which will assist our diagnosis.

In *syphilitic* laryngeal phthisis, the pain is usually much more severe, especially during the act of deglutition, or when pressure is made on the throat, than in the *simple* form. The greater severity of the pain may be owing to the co-existing ulceration of the fauces, or to the presence of venereal vegetations in the pharynx and on the opening of the larynx itself. The mode, in which the disease becomes developed, may also assist eur diagnosis. In the *simple* form, it usually commences in the trachea or larynx, and advances upwards to the epiglottis; whereas in the *venereal* form it most generally proceeds from the fauces downwards.

The *tubercular* laryngeal phthisis is to be distinguished from the other forms of the disease, by the co-existence of those symptoms which are well known to indicate the presence of tubercular disease of the lungs. Hence it is by the stethoscopic signs, by the nature and quantity of the sputa, and by the rapidity of the emaciation, that this form of Laryngeal Phthisis is best discriminated.

In closing these brief remarks, we gladly avail ourselves of the present opportunity to express our high opinion of Mr. Porter's work. It is characterised throughout by great ability, and by the soundest practical judgment. The author evidently writes from experience, and describes what he has seen and observed himself at the bed-side of the sick; and there is altogether such an air of candour in his observations, that the reader at once feels willing to be guided in his practice by them. All physicians and surgeons will therefore do well to add this book to their libraries.

The work of Mr. Ryland is more comprehensive in its scope, professing, as it does, to be a complete treatise on the diseases and injuries of the larynx and trachea, and is founded on an Essay, to which the Jacksonian prize was awarded a few years ago. The author has investigated each subject *seriatim* with praiseworthy diligence, and has availed himself of all the recent continental authorities on pathology, to render his work a text-book on the present state of medical opinion.

